# KLRD1, FOSL2 and LILRB3 as potential biomarkers for plaques progression in acute myocardial infarction and stable coronary artery disease

**DOI:** 10.1186/s12872-021-01997-5

**Published:** 2021-07-16

**Authors:** Qiang Zhang, Yue Zheng, Meng Ning, Tong Li

**Affiliations:** 1 Cardiology, The Third Central Clinical College of Tianjin Medical University, No. 83, Jintang Road, Hedong District, Tianjin, 300170 China; 2 School of Medicine, Nankai University, Tianjin, 300071 China; 3 Cardiology, Nankai University Affiliated Third Center Hospital, Tianjin, 300170 China; 4 Cardiology, The Third Central Hospital of Tianjin, 83 Jintang Road, Hedong District, Tianjin, 300170 China; 5 Tianjin Key Laboratory of Extracorporeal Life Support for Critical Diseases, Tianjin, China; 6 Institute of Hepatobiliary Disease, Tianjin, China

**Keywords:** GEO, WebGestalt, PPI, STEMI, Stable CAD

## Abstract

**Background:**

Myocardial infarction (MI) contributes to high mortality and morbidity and can also accelerate atherosclerosis, thus inducing recurrent event due to status changing of coronary artery walls or plaques. The research aimed to investigate the differentially expressed genes (DEGs), which may be potential therapeutic targets for plaques progression in stable coronary artery disease (CAD) and ST-elevated MI (STEMI).

**Methods:**

Two human datasets (GSE56885 and GSE59867) were analyzed by GEO2R and enrichment analysis was applied through Gene Ontology (GO) and Kyoto Encyclopedia of Genes and Genomes (KEGG) pathway analysis. To explore the seed genes, the protein–protein interaction (PPI) network was constructed and seed genes, as well as top30 ranking neighbours were screened out. To validate these findings, one human dataset GSE120521 was analyzed. Linear regression analysis and ROC curve were also performed to determine which seed genes above mentioned could be independent factors for plaques progression. Mice MI model and ELISA of seed genes were applied and ROC curve was also performed for in vivo validation.

**Results:**

169 DEGs and 573 DEGs were screened out in GSE56885 and GSE59867, respectively. Utilizing GO and KEGG analysis, these DEGs mainly enriched in immune system response and cytokines interaction. PPI network analysis was carried out and 19 seed genes were screened out. To validate these findings, GSE120521 was analyzed and three genes were demonstrated to be targets for plaques progression and stable CAD progression, including KLRD1, FOSL2 and LILRB3. KLRD1 and LILRB3 were demonstrated to be high-expressed at 1d after MI compared to SHAM group and FOSL2 expression was low-expressed at 1d and 1w. To investigate the diagnostic abilities of seed genes, ROC analysis was applied and the AUCs of KLRD1, FOSL2 and LILRB3, were 0.771, 0.938 and 0.972, respectively.

**Conclusion:**

This study provided the screened seed genes, KLRD1, FOSL2 and LILRB3, as credible molecular biomarkers for plaques status changing in CAD progression and MI recurrence. Other seed genes, such as FOS, SOCS3 and MCL1, may also be potential targets for treatment due to their special clinical value in cardiovascular diseases.

**Supplementary Information:**

The online version contains supplementary material available at 10.1186/s12872-021-01997-5.

## Introduction

Cardiovascular diseases are associated with considerable mortality and morbidity. Nowadays acute myocardial infarction (MI) still contributes to the leading mortality in human being [1]. Besides, MI mortality went up by 5.6 times in the last three decades [2]. Previous reports have shown that aged patients with coronary artery disease (CAD) had poorer outcomes, such as higher all‐cause mortality and recurrent event [3, 4]. Early diagnosis of CAD can decrease mortality [5]. So, a deeper understanding of CAD progression may help the diagnosis and treatment, thus saving patients’ lives.

Previous researches reported that MI promoted progenitor cells and haematopoietic stem liberation from bone marrow niches at 1d after MI. The progenitors then seeded the spleen and monocyte production increased, which promoted atherogenesis and therefore contributed to MI progression [6, 7]. Persistent impairment of endothelial vasomotor function was correlated to atherogenesis and plaques progression in ST-elevated myocardial infarction (STEMI) patients’ coronary arteries [8]. Besides, plaque erosion was more frequent in stable CAD, than that in STEMI [9]. So, the biomarkers about patients’ plaques may be novel therapeutic targets for CAD progression and MI recurrence.

In recent years, the potential genes associated with STEMI and stable CAD have been obtained through microarray analysis applied in patients’ peripheral blood and the mice myocardium [10–12]. For example, through the integrated bioinformatics analysis of GEO datasets, Daqiu Chen et al. [11] found 4 hub genes may play a critical role in STEMI development. However, the bioinformatics analysis is rarely used in cardiovascular diseases [13], especially in CAD progression and MI recurrence.

In this study, two human datasets were used to investigate the DEGs. Next, using WebGestalt, GO and KEGG analysis and protein–protein interaction (PPI) network were performed. The seed genes were received for CAD progression and MI recurrence. To validate the seed genes screened in PPI network, one human dataset GSE120521 was analyzed. The linear regression analyses were used to determine which seed genes were independent factors for plaques progression and stable CAD progression. Receiver operating characteristic (ROC) was also applied to evaluate the area under the curve (AUC) value and predictive abilities of these selected genes.

## Methods

### Microarray data

Using the keywords “stable CAD” or “myocardial infarction”, we found two GEO datasets, including GSE56885 contributed by Kapoor et al. and GSE59867 contributed by Maciejak et al. (Table S1). The former was RNA sequencing of human peripheral blood samples in stable CAD patients compared to healthy subjects. The latter was RNA sequencing of human peripheral blood samples in STEMI patients compared to stable CAD. The stable CAD patients were defined as having the disease more than 3 months prior to enrollment and using any combination of cardiac-related medications, for instance, ACEIs, β-blocks and statins. All the patients were angiographically proven.

### Screening for DEGs

To screen out DEGs, the series matrix files were analyzed by applying GEO2R as previously reported [14]. A log_2_FC > 1 and an adjusted *P*-value < 0.05 were applied as the cut-off criteria in GSE56885, while a log_2_FC > 0.38 and an adjusted *P*-value < 0.05 were used in GSE59867.

### Enrichment analysis

KEGG [15] and GO [16] analysis were performed using Over-Representation Analysis or Gene Set Enrichment Analysis methods in WEB-based Gene Set Analysis Toolkit (WebGestalt) as previously reported [17]. A FDR < 0.05 was significant. Redundancy reduction was applied through a weighted set cover.

### PPI network

To identify the interaction of DEGs, PPI network was built using the Network Topology-based Analysis (NTA, Network Retrieval & Prioritization method) [17]. A FDR < 0.05 was significant.

### Validation of the screened genes in GSE120521

To validate the seed genes screened in NTA, one human dataset about the difference between stable and unstable plaques, GSE120521 contributed by Mahmound et al. (Table S1), was analyzed. Plaques were dissected into stable and unstable regions based on macroscopic appearance. Unstable regions were characterised as the visible zone of plaque rupture, and the surrounding abnormal tissue, including obvious calcification and intra-plaque haemorrhage. Stable regions were macroscopically normal adjacent areas.

### MI model construction

Adult experimental C57Bl/6J male mice (6 mice per group, n = 24) were purchased from Charles River (Beijing, China). Mice were maintained in a specific pathogen-free environment with free access to food and water and a 12/12 light–dark cycle. Protocols were approved by Institute of Radiation Medicine, the Chinese Academy of Medical Science, which conform to the Guide for the Care and Use of Laboratory Animals published by the US National Institutes of Health.

MI were induced in adult young (10–11 weeks). Briefly, heart was manually exposed from the 4th intercostal space through inhalation of isoflurane (1.5–2%, MSS-3, England) and the left coronary artery was located, sutured and ligated at a site about 3 mm from its origin, which induced roughly 50% ischemia of the left ventricular in mice. Infarction was considered successful following the visual appearance of pale discoloration and a ST elevation on electrocardiogram. Sham-operated animals underwent the same procedure of MI model without any coronary artery ligation. To reduce mice pain in animal experiments, the animals were euthanized by cervical dislocation after isoflurane anesthesia (5%, MSS-3, England) to collect left ventricular samples to do ELISA analysis.

### Enzyme-linked immunosorbent assay

For further validations, left ventricular samples in border zone and infarcted area were incubated with primary antibodies overnight at 4 °C and then incubated with secondary antibodies for 1 h at room temperature. Primary antibodies against KLRD1 (LS-C34586-250, 1:30,000, LSBio) and FOSL2 (LS-C801443-100, 1:40,000, LSBio) and LILRB3 (LS-C317999-50, 1:40,000, LSBio), were used.

### Statistical analysis

All data are presented as the mean ± SD. Shapiro–Wilk normality test and Weltch t’ test (2 groups) were performed using SPSS 23.0. The linear regression was applied using the forward method to investigate which seed genes could be independent factors for plaques progression and stable CAD progression. ROC was used to evaluate AUC and predictive abilities. A *P* < 0.05 was considered statistically significant.

## Results

### Identification of DEGs in GSE56885 and GSE59867

**Fig. 1:**
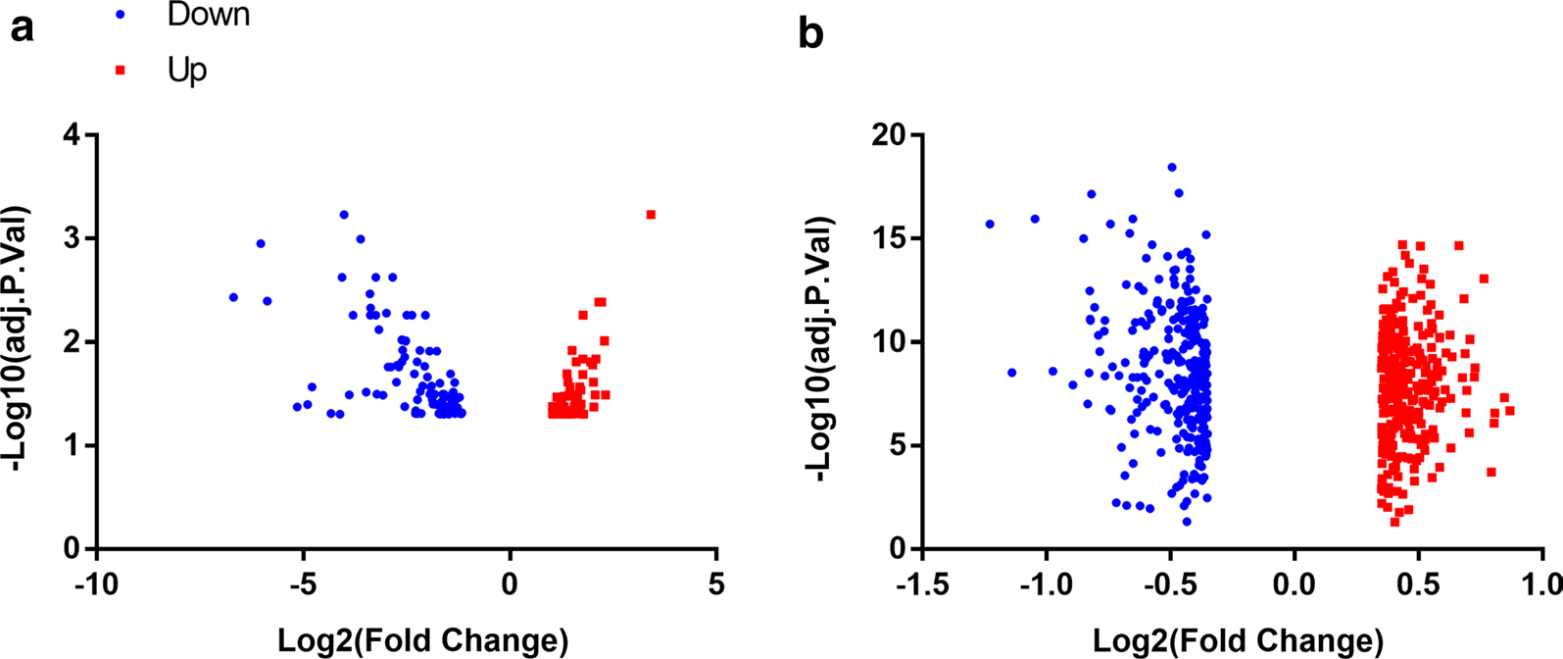
Volcano plot of gene expression profile data in STEMI samples and stable CAD ones. **a** volcano plot of GSE56885, and **b** volcano plot of GSE59867. The red, and blue points represent up-regulated genes and down-regulated genes, respectively. FC, fold change

Two datasets, including GSE56885 and GSE59867, were utilized for analysis. Using GEO2R, 163 DEGs were obtained from GSE56885, including 98 down- and 65 up-regulated genes, while 573 DEGs were obtained from GSE59867, including 284 down- and 289 up-regulated genes (Fig. 1).

### Enrichment analysis of DEGs

**Fig. 2:**
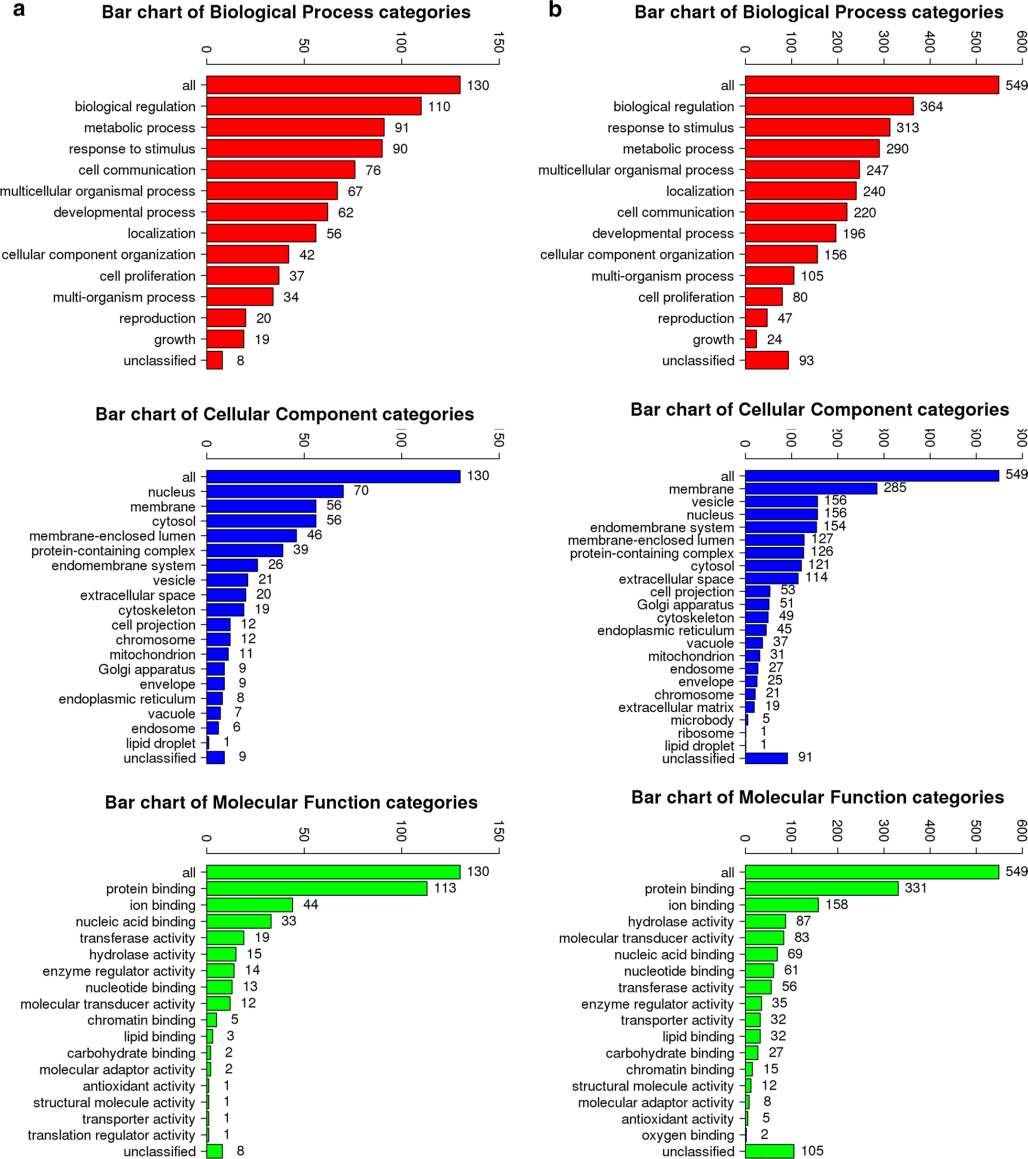
The results of GO enrichment analysis in STEMI samples and stable CAD ones. The results of GO enrichment analysis of GSE56885 (**a**), and GSE59867 (**b**) were shown, including biological process (red), cellular component (blue), and molecular function (green)

**Fig. 3:**
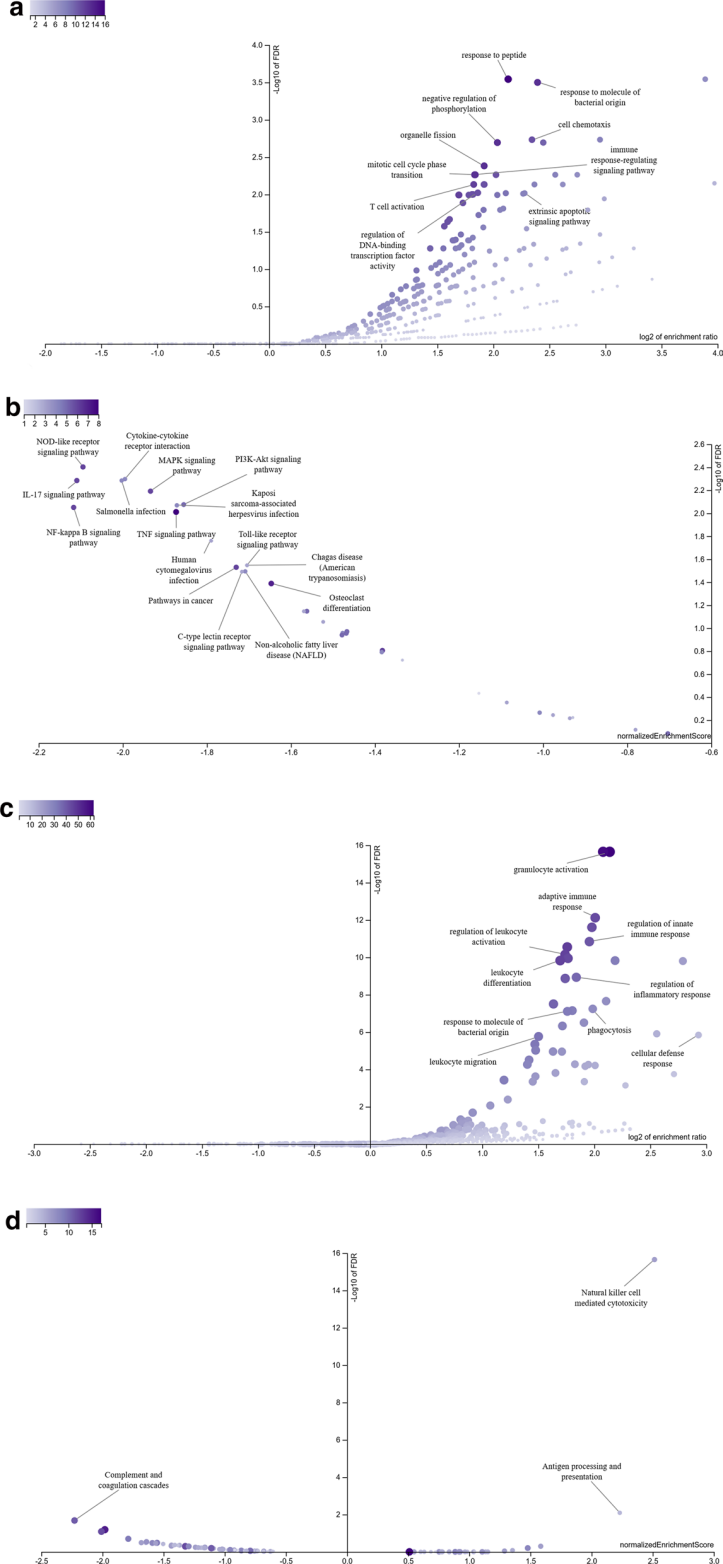
Volcano plot of enriched pathways in STEMI samples and stable CAD ones. **a** The volcano plot of GO enrichment analysis of GSE56885 using ORA method. **b** The volcano plot of KEGG enrichment analysis of GSE56885 using GESA method. **c** The volcano plot of GO enrichment analysis of GSE59867 using ORA method. **d** The volcano plot of KEGG enrichment analysis of GSE59867 using GESA method

Using WebGestalt, GO slim and enrichment analysis were performed. The enriched GO terms were largely identical but with minor differences in two datasets (Fig. 2). Enriched GO terms and ancestor of enriched terms were also shown (Additional file 1: Figure S1). The result of GO analysis demonstrated that the DEGs in GSE56855 were mainly enriched in 10 pathways, for instance, response to peptide and cell chemotaxis, while those in GSE59867 were also mainly involved in 10 pathways, for instance, granulocyte activation and adaptive immune response (Tables 1, 2; Fig. 3a, c).

**Table 1:** The results for GO analysis in stable CAD patients compared to healthy subjects

Gene set	Description	Size	Expect	Ratio	*P* value	FDR
GO:1901652	Response to peptide	487	3.6508	4.3826	6.73E−07	0.00028587
GO:0002237	Response to molecule of bacterial origin	330	2.4738	5.255	0.000001115	0.00031591
GO:0060326	Cell chemotaxis	289	2.1665	5.0774	0.000010863	0.0018467
GO:0042326	Negative regulation of phosphorylation	423	3.171	4.0997	0.000016617	0.0020178
GO:0048285	Organelle fission	459	3.4409	3.7781	0.00003896	0.0041396
GO:0002764	Immune response-regulating signaling pathway	485	3.6358	3.5756	0.000068438	0.0054448
GO:0044772	Mitotic cell cycle phase transition	487	3.6508	3.5609	0.000071351	0.0054448
GO:0042110	T cell activation	452	3.3884	3.5415	0.00014479	0.007344
GO:0051090	Regulation of DNA-binding Transcription factor activity	404	3.0286	3.6321	0.00022327	0.0094891
GO:0097191	Extrinsic apoptotic signaling pathway	220	1.6492	4.8508	0.00024866	0.0096073

GO, gene-ontology; CAD, coronary artery disease; FDR, false discovery rate

**Table 2:** The results for GO analysis in MI patients compared to stable CAD ones

Gene set	Description	Size	Expect	Ratio	*P* value	FDR
GO:0036230	Granulocyte activation	500	14.318	4.4002	0	0
GO:0002250	Adaptive immune response	382	10.939	4.0224	2.66E−15	7.55E−13
GO:0045088	Regulation of innate immune response	369	10.566	3.8802	8.45E−14	1.44E−11
GO:0002694	Regulation of leukocyte activation	481	13.773	3.3398	5.88E−13	7.13E−11
GO:0002521	Leukocyte differentiation	496	14.203	3.2387	1.75E−12	1.49E−10
GO:0050727	Regulation of inflammatory response	361	10.337	3.5793	1.65E−11	1.17E−09
GO:0006909	Phagocytosis	238	6.8152	3.9618	1.08E−09	5.73E−08
GO:0002237	Response to molecule of bacterial origin	330	9.4496	3.3864	1.66E−09	7.84E−08
GO:0006968	Cellular defense response	55	1.5749	7.6194	3.74E−08	1.4469E−06
GO:0050900	Leukocyte migration	419	11.998	2.8338	4.69E−08	1.7328E−06

GO, gene-ontology; CAD, coronary artery disease; FDR, false discovery rate

Using GSEA, no positive related category and 16 negative related categories were identified as enriched categories of KEGG pathway analysis in GSE56855. These genes were mainly involved in the NOD-like receptor signalling pathway, Cytokine-cytokine receptor interaction, IL-17 signalling pathway and PI3K-Akt signalling pathway. Using GSEA, 2 positive related categories and 1 negative related categories were identified as enriched categories in GSE59867. These DEGs were enriched in Natural killer cell-mediated cytotoxicity, Antigen processing and presentation, and Complement and coagulation cascades (Table 3, 4; Fig. 3b, d).

**Table 3:** The results for KEGG analysis in stable CAD patients compared to healthy subjects

Gene set	Description	Size	Leading edge number	ES	NES	*P* value	FDR
hsa04621	NOD-like receptor signaling pathway	8	6	− 0.77061	− 2.0947	0.0014245	0.0039551
hsa04060	Cytokine-cytokine receptor interaction	7	4	− 0.77594	− 1.9948	0.001462	0.0050427
hsa05132	Salmonella infection	5	4	− 0.87092	− 2.0026	0	0.0051911
hsa04657	IL-17 signaling pathway	7	6	− 0.8278	− 2.1093	0	0.0051911
hsa04010	MAPK signaling pathway	7	6	− 0.7715	− 1.9341	0.0014451	0.006427
hsa04151	PI3K-Akt signaling pathway	7	5	− 0.71874	− 1.855	0.005571	0.0084046
hsa05167	Kaposi sarcoma-associated herpesvirus infection	7	4	− 0.72723	− 1.8713	0.0028011	0.0085282
hsa04064	NF-kappa B signaling pathway	8	6	− 0.79671	− 2.1169	0.0013966	0.008899
hsa04668	TNF signaling pathway	9	8	− 0.67798	− 1.8731	0.002751	0.0097465
hsa05163	Human cytomegalovirus infection	5	3	− 0.77931	− 1.7897	0.004644	0.017353
hsa04620	Toll-like receptor signaling pathway	5	3	− 0.74322	− 1.7044	0.01682	0.028378
hsa05142	Chagas disease (American trypanosomiasis)	5	3	− 0.74322	− 1.7044	0.01682	0.028378
hsa05200	Pathways in cancer	7	6	− 0.6674	− 1.7301	0.011561	0.029528
hsa04932	Non-alcoholic fatty liver disease (NAFLD)	5	4	− 0.74563	− 1.7086	0.006006	0.032059
hsa04625	C-type lectin receptor signaling pathway	5	3	− 0.74881	− 1.7165	0.010753	0.032259
hsa04380	Osteoclast differentiation	7	7	− 0.65041	− 1.6467	0.016129	0.04088

KEGG, Kyoto encyclopedia of genes and genomes; CAD, coronary artery disease; FDR, false discovery rate

**Table 4:** The results for KEGG analysis in MI patients compared to stable CAD ones

Gene set	Description	Size	Leading edge number	ES	NES	*P* value	FDR
hsa04650	Natural killer cell mediated cytotoxicity	10	6	0.69503	2.5146	0	0
hsa04612	Antigen processing and presentation	6	4	0.77405	2.2306	0.0020747	0.0081146
hsa04610	Complement and coagulation cascades	12	11	-0.59536	-2.2285	0	0.020906

KEGG, Kyoto encyclopedia of genes and genomes; CAD, coronary artery disease; FDR, false discovery rate

### Detection of the key genes in STEMI and stable CAD

A PPI BioGRID network was created through NTA to detect the seed genes in STEMI and stable CAD. Nine hub genes were screened out in GSE56855 (Additional file 2: Figure S2), such as FOSL2, BCL6, JUNB, and FOS, while 10 hub genes were screened out in GSE59867 (Additional file 3: Figure S3), for instance, FOS, TRIM25, SOCS3, KLRD1 and LILRB3. Besides the seed genes and their top30 ranking neighbours were also shown (Table S2). Three genes, including FOS, BCL6 and SOCS3, were both screened out in two datasets and their expressions were both significantly lower in STEMI and stable CAD patients compared to controls (Additional file 4: Figure S4).

### Validation of the screened genes in GSE120521

**Fig. 4:**
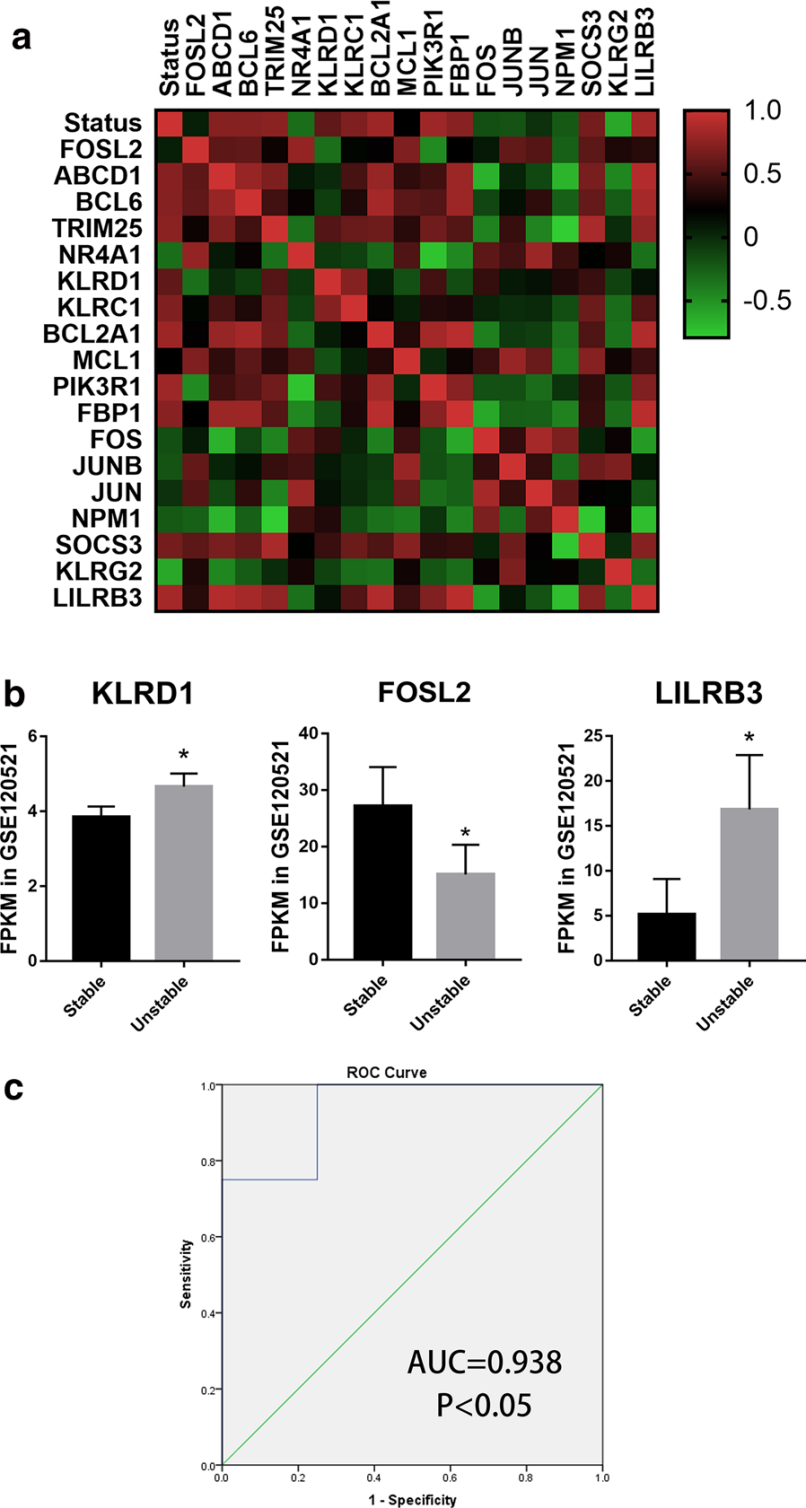
Validation of the screened genes. **a** The correlationship among the plaques status and the screened seed genes in human plaques in unstable region compared to stable region. **b** The three screened genes FPMK value of RNA sequencing in GSE120521. *P < 0.05. **c** The ROC curve analysis of the three‐gene signature (KLRD1 + FOSL2 + LILRB3) for the discrimination of stable and unstable plaques in GSE120521 dataset. AUC indicates area and *P*‐value is shown under the ROC curve, respectively. AUC, area under the curve; ROC, receiver operating characteristic

For the validation of the findings, GSE120521 dataset was analyzed, which included RNA sequencing of stable atherosclerosis plaques and unstable plaques. The linear regression was applied to investigate which seed genes could be independent factors for plaques progression (Table 5; Fig. 4). After regression analysis, three genes were screened, which may be the diagnosis targets for plaques progression. The mRNA expressions of KLRD1 and LILRB3 increased in unstable plaques compared to stable plaques, while the mRNA expressions of FOSL2 decreased (Fig. 4b). The AUC value of three genes combined effect was 0.938 (Fig. 4c, *P* < 0.05), suggesting the three genes may be diagnostic and therapeutic targets for plaques progression.

**Table 5:** The linear regression of seed genes in GSE120521

Model		Unstandardized coefficients		Standardized coefficients	*P* value	95.0% CI		Collinearity statistics	
		B	Std. error	β		Lower bound	Upper bound	Tolerance	VIF
1	(Constant)	0.903	0.221		0.007	0.361	1.445		
	LILRB3	0.054	0.017	0.798	0.018	0.013	0.095	1	1
2	(Constant)	− 0.958	0.474		0.1	− 2.177	0.262		
	LILRB3	0.057	0.009	0.834	0.001	0.034	0.08	0.995	1.005
	KLRD1	0.444	0.11	0.529	0.01	0.162	0.726	0.995	1.005
3	(Constant)	− 2.158	0.33		0.003	− 3.076	− 1.24		
	LILRB3	0.077	0.006	1.124	0	0.061	0.093	0.459	2.18
	KLRD1	0.406	0.049	0.484	0.001	0.27	0.542	0.968	1.033
	FOSL2	0.003	0.001	0.4	0.009	0.001	0.005	0.449	2.228

CI, confidence interval

### Validations of screened seed genes in mouse MI model

**Fig. 5:**
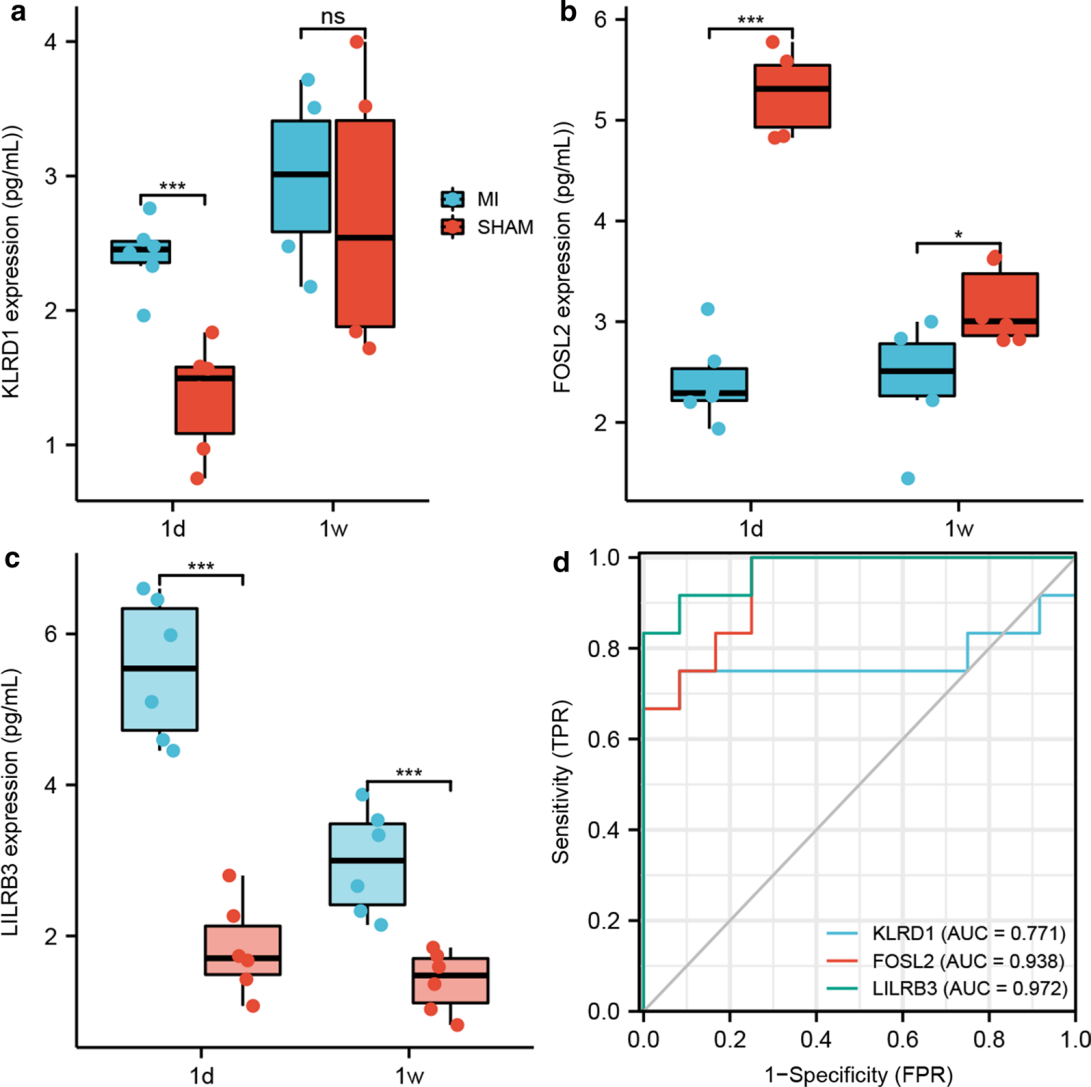
Validations of screened genes. **a**–**c** The protein expression levels of left ventricle at 1d and 1w after MI utilizing ELISA, including KLRD1 (**a**), FOSL2 (**b**) and LILRB3 (**c**). **d** The ROC curve analysis of the three genes in mice MI model. AUC, area under the curve; ROC, receiver operating characteristic. *P < 0.05; ***P < 0.001; NS, not significant

To validate the function of screened seed genes, ELISA of left ventricular was applied in mice MI model. KLRD1 and LILRB3 were demonstrated to be high-expressed at 1d after MI compared to SHAM group, while there was no significant difference of KLRD1 expression at 1w. Besides, FOSL2 expression in border zone was demonstrated to be low-expressed at 1d and 1w after MI compared to SHAM group (Fig. 5a–c).

To investigate the diagnostic abilities of seed genes, ROC analysis was applied. The AUCs of protein, including KLRD1, FOSL2 and LILRB3, were 0.771, 0.938 and 0.972, suggesting KLRD1, FOSL2 and LILRB3 may be the diagnostic and prognostic biomarkers for MI progression and recurrence (Fig. 5d).

## Discussion

Previous researches demonstrated the aetiology of stable CAD and MI, however, the predictive biomarkers and treatment targets are still limited [5, 18]. Previous researches demonstrated that CAD progression, including healthy subjects to stable CAD and stable CAD to STEMI, can induce plaques progression and plaques progression can, in turn, deteriorate CAD progression [6–8]. In the current study, the datasets GSE56885 and GSE59867 were used to screen new potential biomarkers for unstable plaques and CAD progression. The dataset GSE56885 discussed the difference of peripheral blood samples between stable CAD patients and healthy subjects, while the dataset GSE59867 discussed the difference of peripheral blood samples between stable CAD patients and STEMI patients. Because the former expression difference was higher than that of later, the fold change cutoff value of the latter was also lower. Although the GSE59867 dataset contained 436 samples, out of which 390 samples from patients (n = 111) with STEMI at four time points (admission, discharge, 1 month after MI, and 6 months after MI) and 46 samples from patients (n = 46) with stable CAD and without a history of MI were included in the study, we only chose admission patients data with STEMI and stable CAD because MI can accelerate atherosclerosis at 1d after MI, thus inducing recurrent event due to status changing of coronary artery walls or plaques [6]. The dataset GSE12521 was analyzed for the further validation of seed genes. After that, KLRD1, FOSL2 and LILRB3 were demonstrated to serve as a novel biomarker for plaques progression and CAD progression.

In this study, by reanalyzing GSE56885, the results of GO and KEGG analysis demonstrated that with the CAD progression, cell immune response and cytokines interaction activated, mainly due to the progression of the plaque, which is consistent with previous reports [19, 20]. PPI network construction unravelled 9 seed genes using NTA. On the other hand, by reanalyzing GSE59867, the results demonstrated that immune system responses, especially innate immune response, were activated in STEMI patients compared to stable CAD patients, which may be due to the lipid accumulation and further deterioration of coronary vessel status [21–23]. The status can be partly reversed by β-blockers not calcium antagonists [24]. PPI network construction in GSE59867 showed 10 seed genes which can be applied to further validation. After validation, three genes were screened. ROC curve and linear regression demonstrated that the combination of KLRD1, FOSL2 and LILRB3 can be applied as a potential biomarker for CAD progression. The AUC was 0.938, suggesting the clinical value in plaques progression and CAD progression. Then the three genes were also validated in mice MI model, demonstrating that KLRD1 and LILRB3 were high-expressed at 1d after MI and FOSL2 expression was low-expressed at 1d and 1w after MI compared to SHAM group. The AUCs of protein KLRD1, FOSL2 and LILRB3, were 0.771, 0.938 and 0.972, respectively. So, KLRD1, FOSL2 and LILRB3 may be the diagnostic and prognostic biomarkers in MI progression period (from stable CAD to unstable CAD) and MI recurrence for plaques progression.

The mRNA expressions of KLRD1 and LILRB3 increased in unstable plaques compared to stable plaques, while the mRNA expression of FOSL2 decreased. KLRD1 (CD94) forms heterodimers with NKG2 resulting in a receptor complex expressed on NK cells and some CD8^+^ T cells [25]. The mRNA expression of KLRD1 often increased in the inflammatory response, for instance, HIV infection, trachoma, and gut dysbiosis [25–27]. Its expression is always with an elevation of Interleukin(IL)-17 and IL-17-related cytokines expression and inhibited by IL-15 [28]. In this study, the mRNA expressions of KLRD1 raised in STEMI patients compared to stable CAD patients and also increased in unstable plaques compared to stable plaques. Leukocyte immunoglobulin-like receptors (LILRBs), associated with MHC class I and microglobulin, activated the JAK/STAT signalling pathway [29] and regulated the suppressive function and fate of MDSCs [30]. In addition, LILRA3 can also induce Takayasu’s arteritis (*P* < 1 × 10^−5^) [31, 32]. In this study, LILRB3 may also play a critical role in the status changing of coronary artery wall and plaques in atherogenesis and CAD progression. Fos-like antigen 2 (FOSL2), as an AP-1 transcription factor, can promote the progenitor to cardiomyocyte transition [33] and FOSL2 over-expression reversed the miR-155 effects on promoting the persistence of exhausted T cells [34]. FOSL2 was also observed to interact with lipid-metabolism-related gene and Fatty acid elongase 4, thus regulating lipid metabolism [35]. In our study, FOSL2 expression declined in unstable plaques compared to stable plaques, which may be due to the lipid metabolic disturbance and response to cell-mediated immunity.

Other seed genes, such as FOS, SOCS3 and MCL1, should also be mentioned due to their special clinical value in cardiovascular diseases. MI can result in the expression change of early response gene FOS, which might be correlated to the neural activity disorders induced by MI [36, 37]. In this study, FOS was both the seed gene in GSE56885 and GSE59867. In other words, FOS is a key biomarker about neural activity for CAD progression and MI occurrence. The suppressor of cytokine signalling 3 (SOCS3), a negative-feedback regulator of the JAK/STAT signaling [38], was enriched as a seed gene in STEMI patients and as a top30 ranking neighbour in stable CAD patients, which may be associated with platelet activity and inflammation. Platelet-induced SOCS3 expression regulated macrophage reprogramming in plaque by increasing IL-6, IL-1β, TNF-αexpression and declined phagocytic capacity that cannot resolve inflammation and maintain plaque growth. A second cohort also indicated that SOCS1: SOCS3 ratio was associated with inflammation and platelet activity [39]. Therefore, SOCS3 may be a potential diagnosis and treatment target for myocardial injury under stress [40]. In addition, myeloid cell leukaemia sequence 1 (MCL1) is critical for mitochondrial function and autophagy in the heart [41]. In this study, MCL1 was enriched in stable CAD and its expression also elevated in STEMI patients with recurrent events compared to those without recurrent events. Rac1 impeded apoptosis through AKT2/MCL1 and increased cell proliferation through JNK/c-JUN/Cyclin-D1 in myocardial hypoxia [42]. MCL1 can also inhibit the mitochondrial apoptosis and maintain cell viability, however, this process was impeded by FBW7. FBW7 can participate in ROS-induced myocardial injury by degrading MCL1 [43]. Therefore, MCL1 may serve as a biomarker of myocardial cell injury in CAD progression.

There are some limitations which should be mentioned. Firstly, only three seed genes were validated for plaques progression. There may be some false negatives because of the enrichment methods and validation methods. More researches are still needed to proceed with integrated bioinformatic analysis about plaques progression. Secondly, we aimed to investigate the potential targets to status changing of plaques and coronary wall to treat CAD progression and MI recurrence. So, we can only discuss a few significant seed genes and their neighbours in this paper. Maybe we can discuss others later. Lastly, the sample sizes of dataset GSE56885 and GSE120521 were not too large, however, after the calculation of sample sizes, they still met the further enrichment analysis and other statistical methods.

## Conclusions

In conclusion, our study provided bioinformatics analysis of STEMI and stable CAD patients compared to their controls, respectively. The screened seed genes, KLRD1, FOSL2 and LILRB3, have been validated as credible molecular biomarkers for plaques progression and CAD deterioration. Other seed genes, such as FOS, SOCS3 and MCL1, may also be potential targets for treatment due to their special clinical value. To verify the current findings, it is also necessary to perform more experiments.

## Supplementary Information

**Additional file 1**. Enriched GO terms and ancestor of enriched terms in STEMI samples and stable CAD ones. (A) Enriched GO terms and ancestor of enriched terms in GSE56885. (B) Enriched GO terms and ancestor of enriched terms in GSE59867

**Additional file 2**. The PPI network graph of screened seed genes and top ranking neighbours in GSE56885, demonstrated 9 genes were screened through NTA

**Additional file 3**. The PPI network graph of screened seed genes and top ranking neighbours in GSE59867, demonstrated 10 genes were screened through NTA

**Additional file 4**. The Log2(FC) value of three screened key genes, FOS, BGL6 and SOCS3

## Data Availability

Microarray data were used from Gene Expression Omnibus (GEO), including GSE56885, GSE59867 and GSE120521. The datasets generated during the current study are available from the corresponding author on reasonable request.

## Abbreviations

MI: Myocardial infarction
DEGs: Differentially expressed genes
CAD: Coronary artery disease
STEMI: ST-elevated myocardial infarction
GO: Gene ontology
KEGG: Kyoto encyclopedia of genes and genomes
PPI: Protein-protein interaction
ROC: Receiver operating characteristic
AUC: Area under the curve
WebGestalt: WEB-based gene set analysis toolkit
NTA: Network topology-based analysis
LILRBs: Leukocyte immunoglobulin-like receptors
FOSL2: Fos-like antigen 2
SOCS3: Suppressor of cytokine signaling 3
MCL1: Myeloid cell leukemia sequence 1

## References

[1] Naghavi M, Abajobir AA, Abbafati C (2017). Global, regional, and national age-sex specific mortality for 264 causes of death, 1980–2016: a systematic analysis for the Global Burden of Disease Study 2016. Lancet.

[2] Chang J, Liu X, Sun Y (2017). Mortality due to acutemyocardial infarction in China from 1987 to 2014: secular trends and ageperiod-cohort effects. Int J Cardiol.

[3] Rodondi N, Marques-Vidal P, Butler J (2010). Markers of atherosclerosis and inflammation for prediction of coronary heart disease in older adults. Am J Epidemiol.

[4] Roger VL, Go AS, Lloyd-Jones DM (2012). Executive summary: Heart disease and stroke statistics-2012 update: a report from the American Heart Association. Circulation.

[5] Cai Y, Yang Y, Chen X (2016). Circulating ‘lncrna OTTHUMT00000387022’ from monocytes as a novel biomarker for coronary artery disease. Cardiovasc Res.

[6] Dutta P, Courties G, Wei Y (2012). Myocardial infarction accelerates atherosclerosis. Nature.

[7] Marino A, Zhang Yi, Rubinelli L (2019). Pressure overload leads to coronary plaque formation, progression, and myocardial events in ApoE–/– mice. JCI Insight.

[8] Horikoshi T, Obata J-e, Nakamura T (2019). Persistent dysfunction of coronary endothelial vasomotor responses is related to atheroma plaque progression in the infarct-related coronary artery of AMI survivors. J Atheroscler Thromb.

[9] Yamamoto E, Yonetsu T, Kakuta T (2019). Clinical and laboratory predictors for plaque erosion in patients with acute coronary syndromes. J Am Heart Assoc.

[10] Zhang T, Zhao L, Cao X (2014). Bioinformatics analysis of time series gene expression in left ventricle (LV) with acute myocardial infarction (AMI). Gene.

[11] Chen D-Q, Kong X-S, Shen X-B (2019). Identification of differentially expressed genes and signaling pathways in acute myocardial infarction based on integrated bioinformatics analysis. Cardiovasc Ther.

[12] Gao Y, Qi G, Guo L (2016). Bioinformatics analyses of differentially expressed genes associated with acute myocardial infarction. Cardiovasc Ther.

[13] Mo X-G, Liu W, Yang Y (2019). NCF2, MYO1F, S1PR4, and FCN1 as potential noninvasive diagnostic biomarkers in patients with obstructive coronary artery: a weighted gene co-expression network analysis. J Cell Biochem.

[14] Yang D, He Y, Bo Wu (2020). Integrated bioinformatics analysis for the screening of hub genes and therapeutic drugs in ovarian cancer. J Ovarian Res.

[15] Kanehisa M, Goto S (2000). KEGG: Kyoto encyclopedia of genes and genomes. Nucleic Acids Res.

[16] Hulsegge I, Kommadath A, Smits MA (2009). Globaltest and GOEAST: two different approaches for gene ontology analysis. BMC Proc.

[17] Cai G, Yang X, Chen T (2020). Integrated bioinformatics analysis of potential pathway biomarkers using abnormal proteins in clubfoot. PeerJ.

[18] Yang Y, Cai Y, Wu G (2015). Plasma long non-coding rna, coromarker, a novel biomarker for diagnosis of coronary artery disease. Clin Sci.

[19] Ponnuswamy P, Van Vre EA, Mallat Z (2012). Humoral and cellular immune responses in atherosclerosis: spotlight on B- and T-cells. Vasc Pharmacol.

[20] Chistiakov DA, Orekhov AN, Bobryshev YV (2016). Immune-inflammatory responses in atherosclerosis: role of an adaptive immunity mainly driven by T and B cells. Immunobiology.

[21] Colivicchi F, Gulizia MM, Arca M (2020). Lipid lowering treatment and eligibility for PCSK9 inhibition in post-myocardial infarction patients in Italy: insights from two contemporary nationwide registries. Cardiovasc Ther.

[22] Fosshaug LE, Colas RA, Anstensrud AK (2019). Early increase of specialized pro-resolving lipid mediators in patients with ST-elevation myocardial infarction. EBioMedicine.

[23] Nguyen MT, Fernando S, Schwarz N (2019). Inflammation as a therapeutic target in atherosclerosis. J Clin Med.

[24] Sorbets E, Steg PG, Young R (2019). β-blockers, calcium antagonists, and mortality in stable coronary artery disease: an international cohort study. Eur Heart J.

[25] Sperk M, Zhang W, Nowak P (2018). Plasma soluble factor following two decades prolonged suppressive antiretroviral therapy in HIV-1-positive males: a cross-sectional study. Medicine (Baltimore).

[26] Burton MJ, Ramadhani A, Weiss HA (2011). Active trachoma is associated with increased conjunctival expression of IL17A and profibrotic cytokines. Infect Immun.

[27] Yang T, Ahmari N, Schmidt JT (2017). Shifts in the gut microbiota composition due to depleted bone marrow beta adrenergic signaling are associated with suppressed inflammatory transcriptional networks in the mouse colon. Front Physiol.

[28] Ramsborg CG, Papoutsakis ET (2007). Global transcriptional analysis delineates the differential inflammatory response interleukin-15 elicits from cultured human T Cells. Exp Hematol.

[29] Truong AD, Hong Y, Lee J (2019). Chicken novel leukocyte immunoglobulin-like receptor subfamilies B1 and B3 are transcriptional regulators of major histocompatibility complex class I genes and signaling pathways. Asian-Australas J Anim Sci.

[30] Ma Ge, Pan P-Y, Eisenstein S (2011). Paired immunoglobin like receptor-B regulates the suppressive function and fate of myeloid derived suppressor cells. Immunity.

[31] Renauer P, Saruhan-Direskeneli G, Coit P (2015). Genome-wide association study identifies susceptibility loci in IL6, RPS9/LILRB3, and an intergenic locus on chromosome 21q22 in Takayasu’s arteritis. Arthritis Rheumatol.

[32] Renauer P, Sawalha AH (2017). The genetics of Takayasu arteritis. Press Med.

[33] Jahangiri L, Sharpe M, Novikov N (2016). The AP-1 transcription factor component Fosl2 potentiates the rate of myocardial differentiation from the zebrafish second heart field. Development.

[34] Stelekati E, Chen Z, Manne S (2018). Long-term persistence of exhausted CD8 T cells in chronic infection is regulated by MicroRNA-155. Cell Rep.

[35] Li S, Teegarden A, Bauer EM (2017). Transcription factor CTIP1/ BCL11A regulates epidermal differentiation and lipid metabolism during skin development. Sci Rep.

[36] Ahn JY, Tae HJ, Cho JH (2015). Activation of immediate-early response gene c-Fos protein in the rat paralimbic cortices after myocardial infarction. Neural Regen Res.

[37] Roy RK, Augustine RA, Brown CH (2018). Activation of oxytocin neurons in the paraventricular nucleus drives cardiac sympathetic nerve activation following myocardial infarction in rats. Commun Biol.

[38] Nagata T, Yasukawa H, Kyogoku S (2015). Cardiac-specific SOCS3 deletion prevents in vivo myocardial ischemia reperfusion injury through sustained activation of cardioprotective signaling molecules. PLoS ONE.

[39] Barrett TJ, Schlegel M, Zhou F (2019). Platelet regulation of myeloid suppressor of cytokine signaling 3 accelerates atherosclerosis. Sci Transl Med.

[40] Yasukawa H, Nagata T, Oba T (2012). SOCS3: a novel therapeutic target for cardioprotection. JAKSTAT.

[41] Thomas RL, Gustafsson ÅB (2013). MCL1 is critical for mitochondrial function and autophagy in the heart. Autophagy.

[42] Zhao J, Jie Q, Li G (2018). Rac1 promotes the survival of H9c2 cells during serum deficiency targeting JNK/c-JUN/Cyclin-D1 and AKT2/MCL1 pathways. Int J Med Sci.

[43] Li X, Zhang N, Zhang Y (2019). E3 ligase Fbw7 participates in oxidative stress-induced myocardial cell injury via interacting with Mcl-1. Mol Med Rep.

