# Supplementary figures and images for: KLRD1, FOSL2 and LILRB3 as potential biomarkers for plaques progression in acute myocardial infarction and stable coronary artery disease
Source: BMC Cardiovasc Disord. 2021 Jul 16;21:344. doi: 10.1186/s12872-021-01997-5 (PMC8285847; doi:10.1186/s12872-021-01997-5)

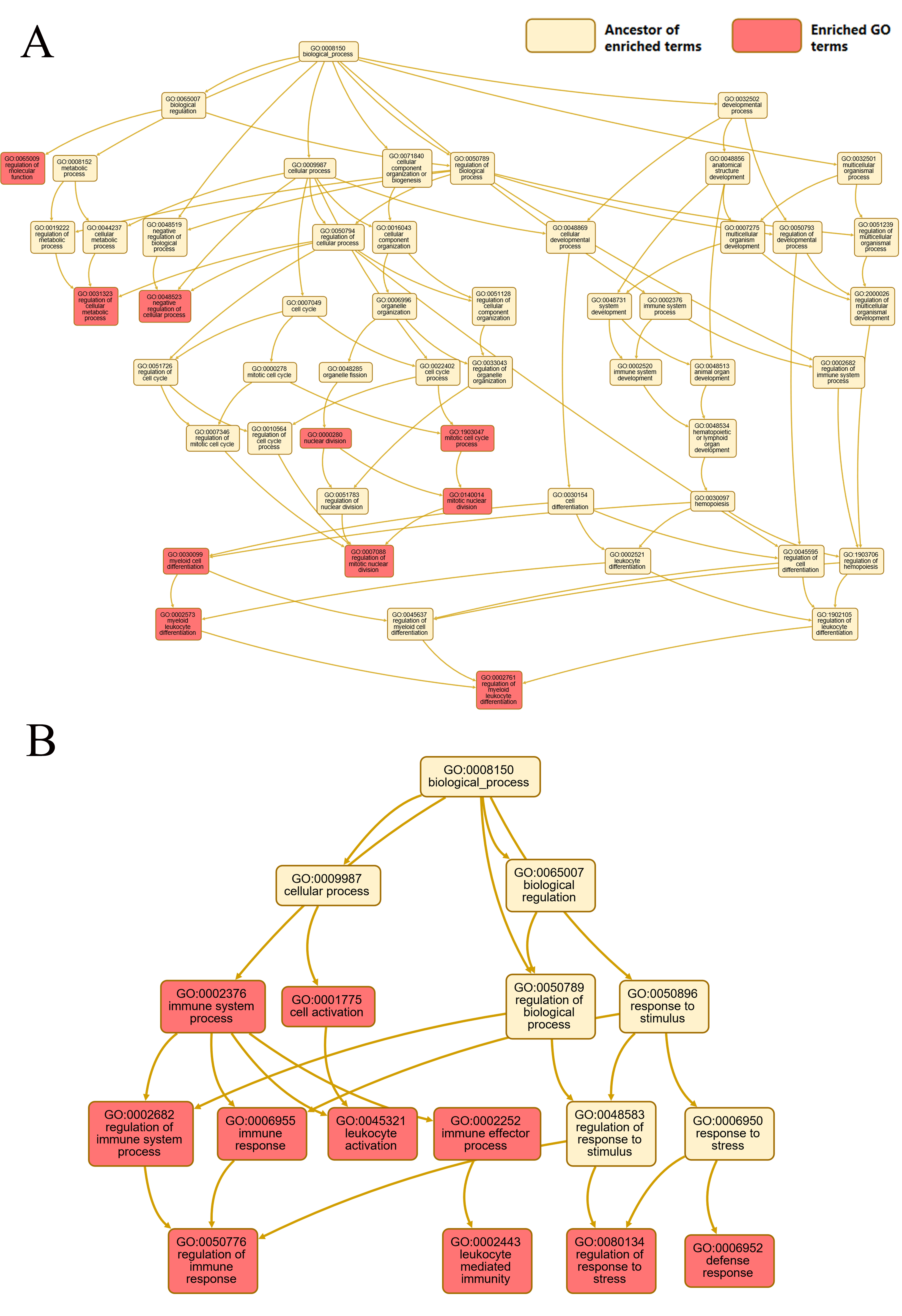

Supplement: Supplementary file 1 — Additional file 1. Enriched GO terms and ancestor of enriched terms in STEMI samples and stable CAD ones. (A) Enriched GO terms and ancestor of enriched terms in GSE56885. (B) Enriched GO terms and ancestor of enriched terms in GSE59867 [file 12872_2021_1997_MOESM1_ESM.tif]

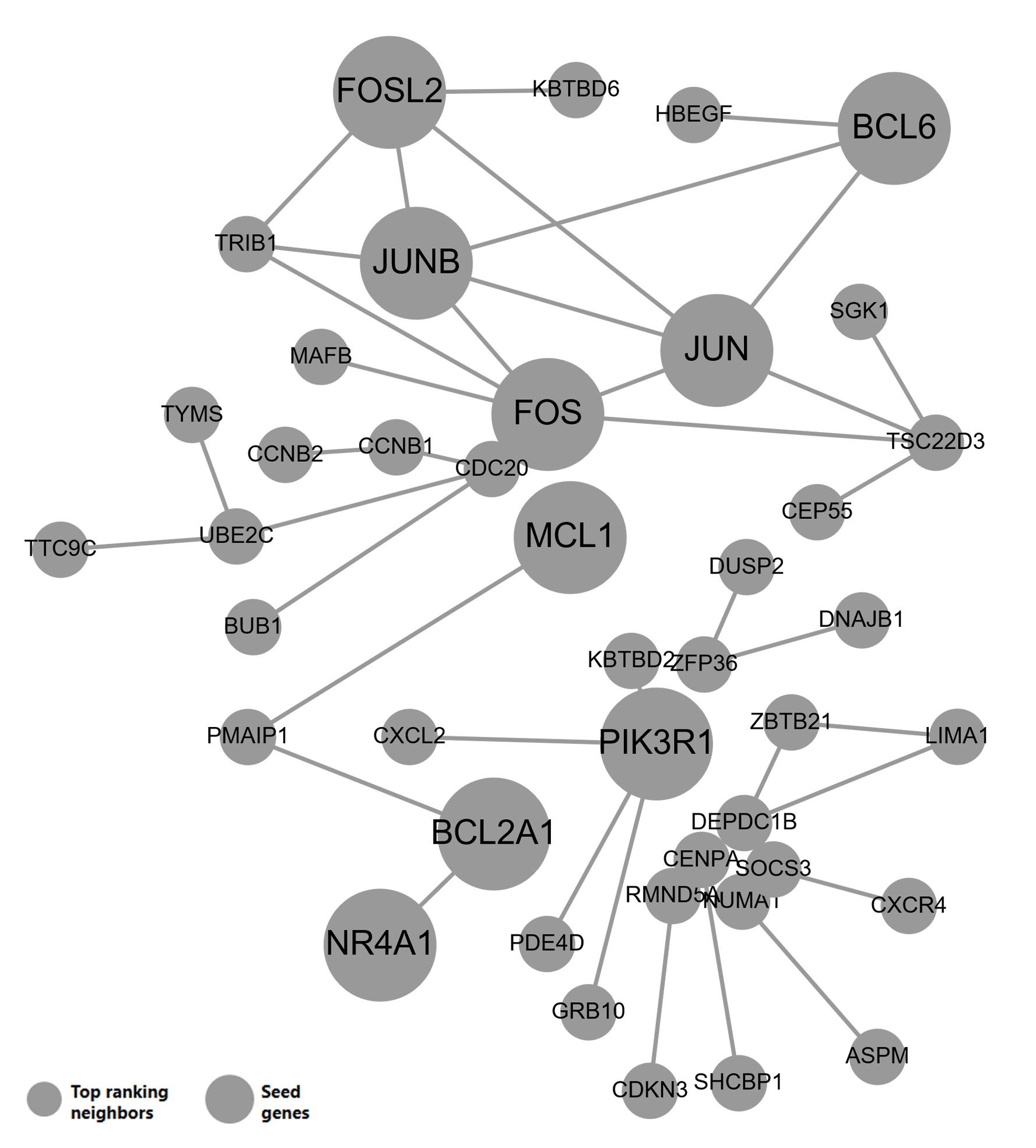

Supplement: Supplementary file 2 — Additional file 2. The PPI network graph of screened seed genes and top ranking neighbours in GSE56885, demonstrated 9 genes were screened through NTA [file 12872_2021_1997_MOESM2_ESM.tif]

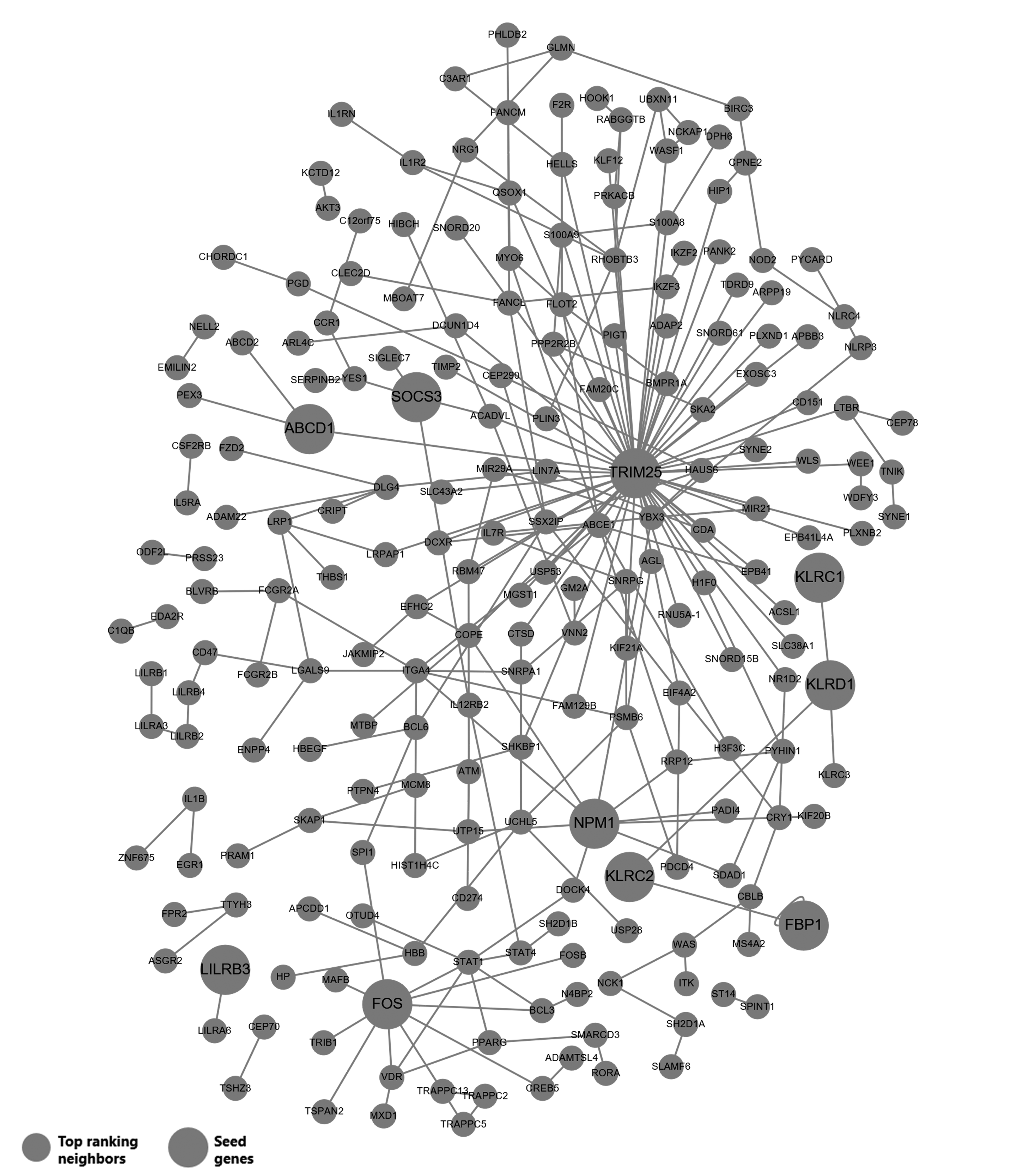

Supplement: Supplementary file 3 — Additional file 3. The PPI network graph of screened seed genes and top ranking neighbours in GSE59867, demonstrated 10 genes were screened through NTA [file 12872_2021_1997_MOESM3_ESM.tif]

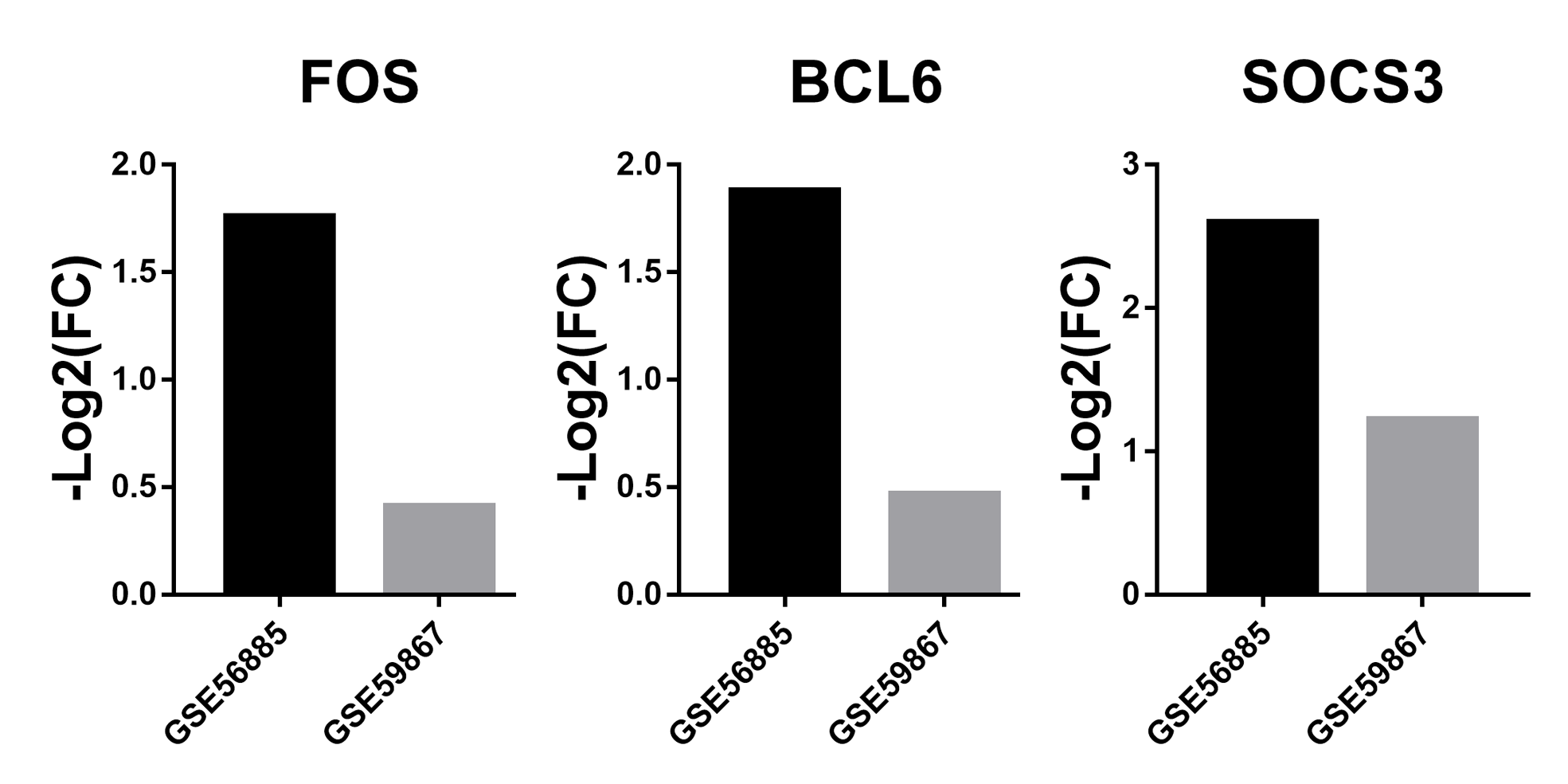

Supplement: Supplementary file 4 — Additional file 4. The Log2(FC) value of three screened key genes, FOS, BGL6 and SOCS3 [file 12872_2021_1997_MOESM4_ESM.tif]
